# Factors Associated With Digital Addiction: Umbrella Review

**DOI:** 10.2196/66950

**Published:** 2025-07-28

**Authors:** Yun Han, Jiamin Qiu, Chengbin Shi, Shiqi Huang, Haokun Huang, Xinman Wang, Sui Zhu, Da-lin Lu, Peng Lu, Fangfang Zeng

**Affiliations:** 1 School of Marxism, Huaqiao University, Xiamen, China; 2 Department of Public Health and Preventive Medicine, School of Medicine, Jinan University, No. 601, Huangpu Avenue West, Tianhe District, Guangzhou, 510632, China, 86 2085221343; 3 Chinese Language and Culture College, Huaqiao University, Xiamen, China

**Keywords:** digital addiction, influencing factor, umbrella review, meta-analysis, youth, adolescents, teenager, urban, AMSTAR 2, GRADE, adverse childhood experiences, social anxiety, psychological, psychology, social problem, social support, methodologies, public health, digital health, mental health

## Abstract

**Background:**

Digital addiction, affecting a significant portion of the population, particularly young people, is linked to psychological issues and social problems, making its prevention and management a crucial public health issue.

**Objective:**

This umbrella review aimed to comprehensively analyze the factors influencing digital addiction by re-evaluating the methodologies and evidence quality of existing meta-analyses.

**Methods:**

Databases including PubMed, Web of Science, the Cochrane Library, and Embase were systematically searched for reviews and meta-analyses related to factors associated with digital addiction up to September 24, 2024. The methodological quality of the identified studies was assessed using the modified “A Measurement Tool to Assess Systematic Reviews 2” (AMSTAR 2) tool, while the Grading of Recommendations Assessment, Development, and Evaluation (GRADE) tool was used to evaluate the quality of the evidence presented in the literature.

**Results:**

A total of 18 articles were included in the review, with AMSTAR 2 assessments revealing 6 moderate-quality, 4 low-quality, and 8 very low-quality studies. In total, 29 significant factors associated with digital addiction were identified. Notably, 4 studies achieved both moderate AMSTAR 2 and GRADE ratings, indicating that positive parent-child relationship (*r*=−0.15, 95% CI−0.18 to −0.11; *P*<.05) effectively mitigate the risks of digital addiction. Conversely, urban residence (odds ratio [OR] 2.32, 95% CI 1.19-4.53; *P*<.001), adverse childhood experiences (*r*=0.21, 95% CI 0.18-0.24; *P*<.001), and social anxiety (*r*=0.34, 95% CI 0.19-0.48; *P*<.001) were identified as factors that may increase the risk of developing digital addiction.

**Conclusions:**

This study found that social factors such as urban residence were associated with an increased risk of digital addiction, whereas social support served as a protective factor against this risk. Nonetheless, the methodologies used in analyzing the factors related to digital addiction require further refinement.

## Introduction

Digital addiction is characterized by a detrimental dependence on digital media and high-tech devices, including social networking, smartphone, and internet addictions [1,2]. Although digital addiction is not officially classified as a diagnosable disorder, some psychologists argue that it bears similarities to substance use disorders [3]. It is estimated that smartphone addiction affected 26.99% of individuals, social media addiction 17.42%, and internet addiction 14.22%, with notably higher rates among young people [2,4]. A cross-sectional study involving 31,659 university students from 2015 to 2018 revealed prevalence rates of 37.93% (12,009/31,659) for mild, 6.33% (2003/31,659) for moderate, and 0.20% (63/31,659) for severe internet addiction [5]. Digital addiction contributes to psychological issues, including attention deficits, depression, and anxiety, while also exacerbating social isolation, sleep disorders, and diminishing academic and occupational performance [6]. Consequently, the effective prevention and management of digital addiction has emerged as a critical public health concern [7].

Studies have sought to identify modifiable risk and protective factors associated with digital addiction, categorizing them into three main domains, namely individual, digital media, and environment [7-11]. The emphasis has primarily been on individual and social determinants, which encompass intrapersonal and interpersonal factors, including gender differences, psychological traits, and health behaviors, as well as broader social influences. Despite the existence of numerous systematic reviews and meta-analyses addressing factors linked to digital addiction, these investigations often focus on specific individual factors without providing updated, comprehensive insights. It is notable that 1 study identified 34 modifiable factors such as intrapersonal factors (eg, time spent playing and psychological conditioning), interpersonal factors, and social factors and concluded that intrapersonal factors were more likely to have a greater impact on internet gaming disorder [8]. However, this study’s focus on internet gaming disorder represents only 1 facet of the broader spectrum of digital addiction.

Umbrella review is an emerging evidence synthesis method that evaluates multiple meta-analyses on related topics to derive more comprehensive conclusions. Unlike traditional meta-analyses, umbrella reviews assess the methodological quality and risk of bias across various studies, enhancing the ability to detect inconsistencies and differences in findings [12]. This approach offers a broader understanding of the credibility and robustness of the evidence, helping to identify potential heterogeneity and diversity among studies.

This study aimed to further promote the integration of different research findings by conducting a comprehensive analysis of existing influences on digital addiction through umbrella analyses, while also evaluating the methodology and quality of existing meta-analyses and systematic reviews.

## Methods

### Study Design

This umbrella review was conducted in accordance with the registered protocol (Open Science Framework Registry) and the PRISMA (Preferred Reporting Items for Systematic Reviews and Meta-Analyses) 2020 statement [13]. Our study protocol has been registered with PROSPERO (registration number CRD42024594911).

### Literature Search Strategy

This study followed the umbrella review process established by Aromataris et al [12], searched databases such as PubMed, Web of Science, the Cochrane Library, and Embase, and selected systematic reviews and meta-analyses on the influencing factors of digital addiction included in the databases up to September 24, 2024. The keywords used included digital addiction, digital overuse, internet addiction, internet-based addiction, problematic internet use, gaming disorder, social media addiction, influencing factors, risk factors, causative factors, contributing factors, meta-analysis, and systematic review, etc, and the search was conducted using a combination of subject words and free words. Taking the PubMed database as an example, the search strategy was shown in Multimedia Appendix 1. We also hand-searched the references of eligible included studies and google scholar to identify further relevant articles.

### Inclusion and Exclusion Criteria

We included studies on the influencing factors of digital addiction if they met the following inclusion criteria: (1) study type was systematic reviews and meta-analyses; (2) studies that focused on the various determinants of any type of digital addiction across different populations; (3) studies that reported the overall effect size, including but not limited to pooled Pearson correlation coefficients or Spearman rank correlation coefficients (*r*), standardized mean difference, and odds ratio (OR) along with their 95% CIs or credible intervals.

Meta-analyses focusing exclusively on a single influencing factor were selected, prioritizing the most recent study with the largest number of included studies when duplicates were present [14]. When multiple systematic reviews addressed the same influencing factor, only the most recent review with the largest number of individual studies providing study-level estimates was included to prevent sample duplication. No language or date restrictions were applied. Articles lacking study-level effect sizes and 95% CIs for systematic reviews, or those based on animal models, were excluded. In addition, we excluded conference abstracts, editorials, narrative reviews, and systematic review protocols.

### Literature Screening and Data Extraction

This study was conducted by 2 researchers who screened the literature and extracted data according to the inclusion and exclusion criteria. In case of disagreement, consulting a third researcher (ZFF) assisted in making the decision. Duplicate literatures were eliminated through the title and abstract, and the full texts were carefully read to extract information such as the first author, publication time, number of studies included and sample size, exposure factors, main outcome indicators, meta-analysis effect model, effect size and its 95% CI. When the same meta-analysis contained multiple health outcome indicators, each outcome indicator was extracted separately.

### Literature Quality Assessment

In this study, the modified version of the systematic review methodology quality assessment tool (AMSTAR 2 [A Measurement Tool to Assess Systematic Reviews 2]) was used to assess the methodological quality of systematic studies [15]. AMSTAR 2 evaluated the methodological quality and credibility of the study from 16 items in 3 dimensions (including 7 key items and 9 non–key items) such as research design and registration, literature search and selection, quality assessment of the study, and data extraction. It demonstrated good inter-rater consistency, practicality, and a wide range of applications. Referencing the interpretation by Zeng et al [16], it was considered that ≤1 non–key item not met was high-quality research, ≥2 non–key items not met was medium-quality research, 1 key item not met was low-quality research, and ≥2 key items not met was extremely low-quality research.

The Grading of Recommendations Assessment, Development, and Evaluation (GRADE) tool was used to evaluate the evidence quality level of the literature [17]. The GRADE grading system evaluated the outcome indicators based on 5 downgrade factors such as limitations, heterogeneity, indirectness, imprecision, and publication bias. This study stipulated that no downgrade factors were high-quality evidence, the existence of 1 downgrade factor was medium-quality evidence, the existence of 2 downgrade factors was low-quality evidence, and downgrade factors ≥3 were extremely low-quality evidence [18,19]. While the GRADE system typically assigns an initial “very low” quality rating to evidence derived from observational studies due to inherent limitations such as potential confounding and bias, it allows for upgrading or downgrading based on additional factors such as the magnitude of effect, dose-response relationships, and the presence of plausible confounding factors. In this umbrella review, we applied the GRADE framework to evaluate the quality of evidence across meta-analyses, which often include a mix of observational and other study designs. This approach enables a more nuanced assessment of the evidence, considering factors such as risk of bias, inconsistency, indirectness, imprecision, and publication bias. By doing so, we aimed to provide a comprehensive evaluation of the credibility and robustness of the findings related to digital addiction.

### Data Analysis

This study conducted a methodological assessment using AMSTAR 2 and evaluated the quality of evidence with GRADE, aiming to identify high-quality and significant results from the most up-to-date and comprehensive available evidence. Significant heterogeneity was defined as present when the *I²*>50% or the *P*<.1 [20]. A meta-analysis was considered to have a small sample size if there were fewer than 1000 participants in the meta-analysis [21,22]. Notably, a significant publication bias was inferred if the largest weighted effect size in the meta-analysis was subordinate to the overall effect size, indicating a significant small study effect (SSE) [21,23]. Publication bias was quantitatively assessed using the Egger test [24] and the Begg test [25], with a *P* value <.05 [26] indicating the presence of statistically significant bias.

## Results

### Literature Screening Results and Basic Characteristics

**Figure 1.**
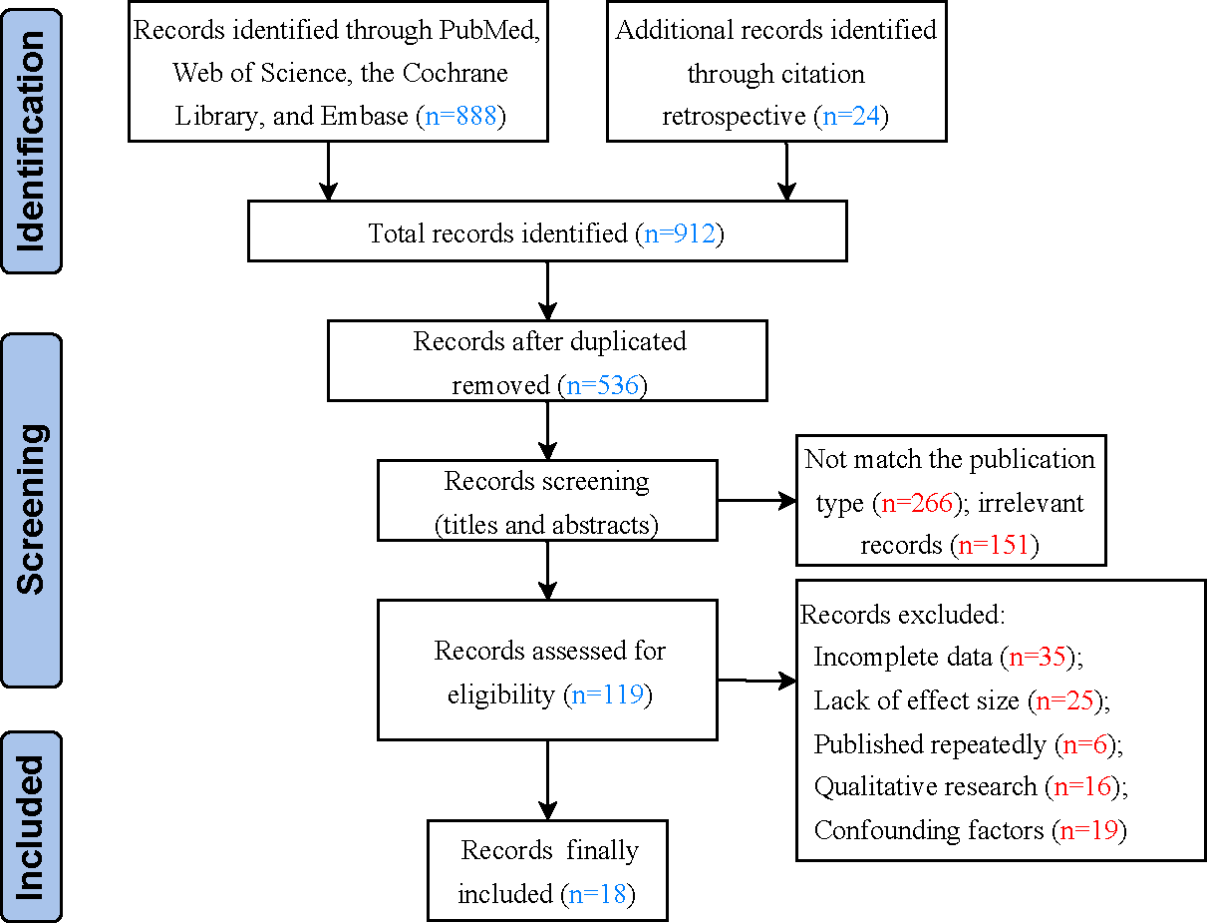
PRISMA flow diagram of the search and selection process.

Through a database search and additional manual search, a total of 912 relevant articles were initially identified. After removing duplicates, 536 articles remained. Following a review of titles and abstracts, 387 articles were excluded (266 due to mismatched study types and 151 for being irrelevant). After full-text screening and reviewing in Multimedia Appendix 1, 101 articles were further excluded due to incomplete data or lack of effect size. As illustrated in Figure 1, the final selection process resulted in the inclusion of 18 articles that met the inclusion criteria.

The included meta-analyses were published between 2020 and 2024, reporting on 24 potential influencing factors related to digital addiction, with personality traits further subdivided into 8 categories. The sample sizes of the studies ranged from 300 to 159,425 participants. The included literature mainly used the New Castle-Ottawa scale (NOS) and the Joanna Briggs Institute (JBI) systematic review checklist to assess research quality. A total 6 studies did not specify the methods used for quality evaluation or risk of bias assessment. The basic characteristics of the included literatures are seen in Table 1.

**Table 1.** The basic characteristics of the studies.

Study	Region	Study design of included study	Studies	Cases	Influencing factor	Outcomes	Quality assessment	I2 values (%)	*P* value of publication bias	SSE^a^
Akbari et al [27]	Iran	Case-control, cohort, experimental study, cross-sectional	85	55,134	Fear of missing out	Internet addiction	—^b^	58.37	0.23	No
Chung et al [7]	Taiwan (China)	—	5	300	Probability discounting	Internet gaming disorder	—	85.66	<.01	Yes
Mak et al [28]	Korea	—	37	34,438	Neuroticism	Facebook addiction	—	96	>.05	Yes
Noroozi et al [29]	Iran	Cross-sectional, case-control, and cohort	18	2589	Quality of life	Internet addiction	STROBE^c^	85.23	0.6	Yes
Bäcklund et al [30]	Sweden	Cross-sectional, longitudinal	46	49,192	Gaming motivations	Gaming disorder	JBI checklist^d^	96	ND	No
Ding et al [31]	China	Cross-sectional	14	11,561	Self-esteem	Smartphone addiction	JBI checklist	74	0.9	No
Eirich et al [32]	Canada	Observational, experimental	87	159,425	Behavior problems	Screen time overuse	JBI checklist	87.8	ND	No
Hao et al [33]	China	Case-control, cross-sectional	26	14,638	Personal relationship	Internet addiction	NOS^e^	94.1	>.05	Yes
Li et al [34]	China	—	50	38,488	Academic burnout	Problematic mobile phone use	—	91.71	0.83	No
Rajesh et al [35]	India	—	96	35,608	Personality traits	Facebook addiction	—	84.27	>.05	No
Wang et al [36]	China	Observational	31	29,692	Negative life events	Internet addiction	NOS	97.8	0.36	No
Zewde et al [37]	Ethiopia	—	145	15,316 7234	Male Urban residence	Internet addiction Internet addiction	— —	<0.01 51.81	.82 .94	Yes No
Hidalgo-Fuentes et al [38]	Spain	Original empirical, quantitative cross-sectional, longitudinal	19	93,859	Resilience	Problematic internet use	NOS	97.46	0.76	No
Niu et al [39]	China	—	3424	32,587 21,366	Positive parenting styles Negative parenting styles	Problematic internet use Problematic internet use	NOS NOS	41.77 30.43	.45 .39	No No
Wan et al [40]	China	Observational	9229	59,716 21,844	Social support Self-control	Mobile phone addiction Smartphone addiction	JBI checklist JBI checklist	96.10 96.30	.81 .19	No No
Zhuang et al [8]	China	Longitudinal, prospective, cohort	12 7 10 5	9113 8444 9364 5819	Depression Gaming time Parent-child relationship Grade point average	Internet gaming disorder Internet gaming disorder Internet gaming disorder Internet gaming disorder	NOS NOS NOS NOS	89.32 97.07 62.54 92.46	.58 .80 .34 .47	No No No No
Hao et al [41]	China	Longitudinal, cross-sectional	37	45,364	Childhood adverse experiences	Internet addiction	NOS	91	>.05	No
Wu et al [42]	China	—	53	59,928	Social anxiety	Problematic social media use	The Agency for Healthcare Research and Quality’s Research Literature Quality Assessment Inventory	92.91	<.01	No

a SSE: small study effect.

b Not mentioned.

c STROBE: Strengthening the Reporting of Observational Studies in Epidemiology.

d JBI checklist: Joanna Briggs Institution Critical Appraisal Checklist.

e NOS: Newcastle-Ottawa scale.

### Methodological Quality Evaluation

Through evaluation with the AMSTAR-2 tool, all included meta-analyses exhibited some methodological deficiencies. Specifically, 6 were of moderate quality, 4 of low quality, and 8 of very low quality, with none classified as high quality. For key items, the compliance rates were as follows: item #2 (whether the review reported that its methods were established a priori and explained any major changes to the protocol), item #4 (whether a comprehensive literature search was conducted), item #7 (whether a list of excluded studies and reasons for exclusion were provided), and item #9 (whether a satisfactory technique for assessing the risk of bias in individual studies was used) all had a compliance rate of 83.33% (15/18); the compliance rate for item #11 (whether appropriate methods were used to combine results) was 72.22% (13/18); item #13 (whether bias was explained) had a compliance rate of 33.33% (6/18); and item #15 (whether the authors properly investigated and discussed the potential impact of publication bias) was 55.56% (10/18). The results of the methodological quality evaluation are presented in Table 2.

**Table 2.** Methodological quality evaluation.

Study	Items																Methodological quality
	#1^a^	#2^b^	#3^c^	#4^d^	#5^e^	#6^f^	#7^g^	#8^h^	#9^i^	#10^j^	#11^k^	#12^l^	#13^m^	#14^n^	#15^o^	#16^p^	
Akbari et al [27]	Y^q^	Y	Y	Y	Y	N^r^	Y	Y	Y	N	N	Y	N	Y	Y	N	Very low
Chung et al [28]	Y	Y	Y	Y	N	Y	Y	Y	Y	Y	Y	Y	N	Y	N	N	Low
Mak et al [29]	Y	N	Y	Y	Y	Y	Y	Y	Y	N	N	N	N	Y	N	N	Very low
Noroozi et al [30]	Y	Y	N	N	N	Y	N	Y	Y	N	Y	N	N	N	N	N	Very low
Bäcklund et al [31]	Y	Y	N	PY^s^	Y	Y	Y	PY	N	Y	N	Y	N	N	Y	Y	Very low
Ding et al [32]	Y	Y	Y	Y	Y	Y	Y	Y	Y	Y	Y	Y	Y	N	Y	N	Moderate
Eirich et al [33]	Y	Y	Y	N	N	Y	Y	Y	Y	Y	Y	N	N	N	N	N	Very low
Hao et al [34]	Y	N	Y	Y	Y	Y	N	Y	Y	Y	Y	N	N	Y	N	N	Very low
Li et al [35]	Y	N	N	Y	N	N	Y	Y	N	Y	N	Y	N	Y	N	N	Very low
Rajesh et al [36]	Y	Y	Y	Y	N	Y	Y	Y	Y	N	Y	Y	N	Y	Y	N	Low
Wan et al [37]	Y	Y	Y	Y	Y	Y	Y	Y	Y	N	Y	N	Y	N	Y	N	Moderate
Wang et al [38]	Y	Y	Y	PY	Y	Y	Y	Y	Y	Y	Y	Y	N	N	Y	N	Low
Zewde et al [39]	Y	Y	Y	Y	Y	N	Y	Y	Y	Y	Y	Y	N	Y	Y	N	Moderate
Hidalgo-Fuentes et al [40]	Y	Y	Y	Y	Y	Y	Y	Y	N	Y	Y	Y	N	Y	N	N	Very low
Niu et al [41]	Y	Y	Y	Y	Y	Y	Y	N	PY	Y	N	Y	Y	Y	Y	N	Low
Zhuang et al [8]	Y	Y	Y	Y	Y	Y	Y	N	Y	Y	Y	Y	Y	Y	Y	N	Moderate
Hao et al [42]	Y	Y	Y	Y	Y	Y	Y	Y	Y	Y	Y	Y	Y	N	N	Y	Moderate
Wu et al [43]	Y	Y	Y	Y	Y	Y	PY	Y	Y	Y	Y	Y	Y	Y	Y	Y	Moderate

a #1-Did the research questions and inclusion criteria for the review include the components of PICO?

b #2-Did the report of the review contain an explicit statement that the review methods were established prior to the conduct of the review and did the report justify any significant deviations from the protocol?

c #3-Did the review authors explain their selection of the study designs for inclusion in the review?

d #4-Did the review authors use a comprehensive literature search strategy?

e #5-Did the review authors perform study selection in duplicate?

f #6-Did the review authors perform data extraction in duplicate?

g #7-Did the review authors provide a list of excluded studies and justify the exclusions?

h #8-Did the review authors describe the included studies in adequate detail?

i #9-Did the review authors use a satisfactory technique for assessing the risk of bias (RoB) in individual studies that were included in the review?

j #10-Did the review authors report on the sources of funding for the studies included in the review?

k #11-If meta-analysis was performed did the review authors use appropriate methods for statistical combination of results?

l #12-If meta-analysis was performed, did the review authors assess the potential impact of RoB in individual studies on the results of the meta-analysis or other evidence synthesis?

m #13-Did the review authors account for RoB in individual studies when interpreting/discussing the results of the review?

n #14-Did the review authors provide a satisfactory explanation for, and discussion of, any heterogeneity observed in the results of the review?

o #15-If they performed quantitative synthesis did the review authors carry out an adequate investigation of publication bias (small study bias) and discuss its likely impact on the results of the review?

p #16-Did the review authors report any potential sources of conflict of interest, including any funding they received for conducting the review?

q Y: complaint.

r N: not complaint.

s PY: partially complaint.

### Evidence Quality Grading

Based on the GRADE evaluation system, of the 24 outcome indicators, 1 study provided high-quality evidence, 5 provided moderate-quality evidence, 11 were of low quality, and 7 were of very low quality. The detailed results of the evidence quality assessment are presented in Table 3.

**Table 3.** Evidence quality grading.

		Downgrading factors					
Study	Influencing Factor	Risk of Bias^a^	Inconsistency^b^	Indirectness^c^	Imprecision^d^	Publication Bias^e^	Evidence Quality
Akbari et al [27]	Fear of missing out	Y^f^	Y	Y	N^g^	N	Very low
Chung et al [28]	Probability discounting	N	N	N	N	N	High
Mak et al [29]	Personality traits	N	Y	N	N	Y	Low
Noroozi et al [30]	Quality of life	Y	Y	Y	Y	Y	Very low
Bäcklund et al [31]	Gaming motivations	Y	N	Y	Y	N	Very low
Ding et al [32]	Self-esteem	N	Y	N	N	Y	Low
	Self-control	Y	Y	Y	Y	Y	Very low
Eirich et al [33]	Behavior problems	Y	N	Y	Y	N	Very low
Hao et al [34]	Personal relationship	N	Y	N	N	Y	Low
Li et al [35]	Academic burnout	N	Y	N	Y	N	Low
Rajesh et al [36]	Personality traits	N	N	N	N	Y	Moderate
Wan et al [37]	Social support	Y	N	Y	N	N	Low
Wang et al [38]	Negative life events	N	Y	N	N	Y	Low
Zewde et al [39]	Gender	N	Y	N	N	Y	Low
	Urban residence	N	N	Y	N	N	Moderate
Hidalgo-Fuentes et al [40]	Resilience	Y	Y	Y	Y	Y	Very low
Niu et al [41]	Positive parenting styles	N	Y	N	Y	N	Low
	Negative parenting styles	N	Y	N	Y	N	Low
Zhuang et al [8]	Depression	N	Y	N	N	Y	Low
	Gaming time	Y	Y	Y	Y	Y	Very low
	Parent-child relationship	N	Y	N	N	N	Moderate
	Grade point average	N	Y	Y	N	N	Low
Hao et al [42]	Childhood adverse experiences	Y	N	N	N	N	Moderate
Wu et al [43]	Social anxiety	N	N	N	N	Y	Moderate

a Risk of bias: bias occurs when the results of a study do not represent the truth because of inherent limitations in design or conduct of a study

b Inconsistency: this refers to unexplained variability among the results of the included studies.

c Indirectness: it refers to the extent to which the people, interventions, and outcome measures are similar to those of interest.

d Imprecision: the authors have the option of decreasing their level of certainty one or two levels if the confidence intervals are wide.

e Publication Bias: this occurs when the dissemination of research findings is influenced by the nature and direction of results.

f Y: compliant.

g NY: not compliant.

### Influencing Factors for Digital Addiction

#### Intrapersonal Factors

**Figure 2.**
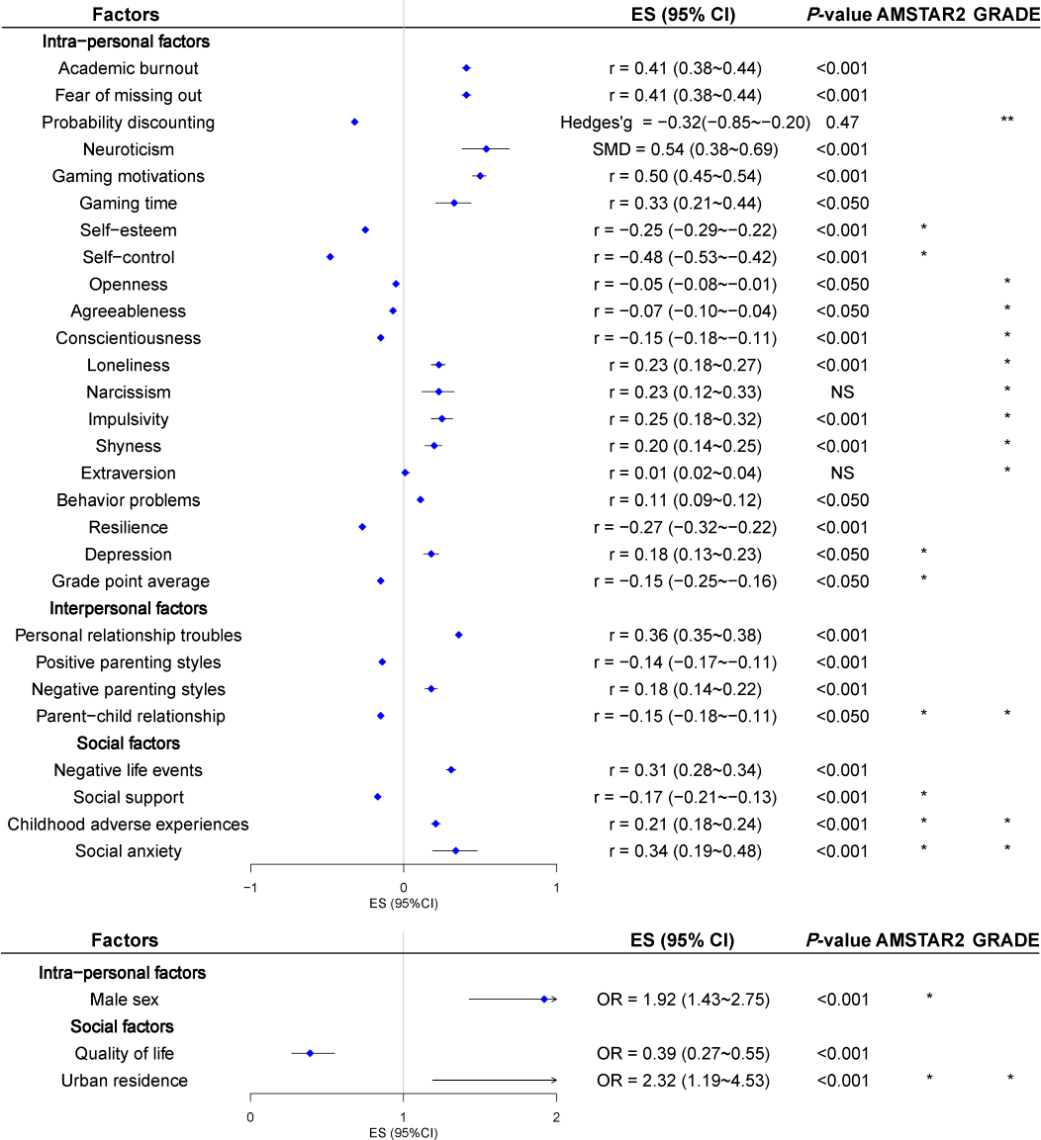
Forest plot of factors associated with digital addiction. AMSTAR 2: a measurement tool to assess systematic reviews version 2; ES: effect size; g: Hedge’s g; GRADE: Grading of Recommendations Assessment, Development, and Evaluation; NS: not significant; OR: odds ratio; *: classified as moderate with AMSTAR 2 or GRADE; **: classified as high with AMSTAR 2 or GRADE.

As shown in Table 4 and Figure 2, most of the included studies were highly significant. In this study, Ding et al [32] discovered that individuals with higher levels of self-esteem (*r*=−0.25, 95% CI−0.29 to −0.22; *P*<.001) and self-control (*r*=−0.48, 95% CI−0.53 to −0.42; *P*<.001) are more prone to digital addiction. Similarly, Zewde et al [39] found that men are at a higher risk of digital addiction compared with women (OR 1.92, 95% CI 1.43-2.75; *P*<.001). Furthermore, Zhuang et al [8] reported that individuals with a higher grade point average (*r*=−0.15, 95% CI −0.25 to −0.16; *P*<.05) are less likely to be threatened by digital addiction. These findings were rated as moderate evidence in terms of methodological quality with AMSTAR 2 tool.

**Table 4.** Factors associated with digital addiction.

Author (Year)	Influencing factor	Model	Confounding adjusted	ES^a^ (95% CI)	*P* value
Intrapersonal factors					
Akbari et al [27]	Fear of missing out	Random	No	*r*=0.41 (0.38-0.44)	<.001
Li et al [35]	Academic burnout	Random	Yes	*r*=0.41 (0.38-0.44)	<.001
Chung et al [28]	Probability discounting	Random	Yes	Hedges *g*=−0.32 (–0.85 to −0.20)	.470
Mak et al [29]	Neuroticism	Random	No	SMD=0.54 (0.38-0.69)	<.001
Bäcklund et al [31]	Gaming motivations	Random	No	*r*=0.50 (0.45-0.54)	<.001
Ding et al [32]	Self-esteem Self-control	Random Random	Yes Yes	*r*=−0.25 (−0.29 to −0.22) *r*=−0.48 (−0.53 to −0.42)	<.001 <.001
Rajesh et al [36]	Openness Agreeableness Conscientiousness Loneliness Narcissism Impulsivity Shyness Extraversion	Random Random Random Random Random Random Random Random	Yes Yes Yes Yes Yes Yes Yes Yes	*r*=−0.05 (−0.08 to −0.01) *r*=−0.07 (−0.10 to −0.04) *r*=−0.15 (−0.18 to −0.11) *r*=0.23 (0.18-0.27) *r*=0.23 (0.12-0.33) *r*=0.25 (0.18-0.32) *r*=0.20 (0.14-0.25) *r*=0.01 (0.02-0.04)	<.05 <.05 <.001 <.001 NS^b^ <.001 <.001 NS
Eirich et al [33]	Behavior problems	Random	Yes	*r*=0.11 (0.09-0.12)	<.05
Zewde et al [39]	Male sex	Fix	No	OR^c^ 1.92 (1.43-2.75)	<.001
Hidalgo-Fuentes et al [40]	Resilience	Random	No	*r*=−0.27 (−0.32 to −0.22)	<.001
Zhuang et al [8]	Depression Grade point average Gaming time	Random Random Random	No No No	*r*=0.18 (0.13-0.23) *r*=−0.15 (−0.25 to −0.16) *r*=0.33 (0.21-0.44)	<.05 <.05 <.05
Interpersonal factors					
Hao et al [34]	Personal relationship troubles	Random	No	*r*=0.36 (0.35-0.38)	<.001
Niu et al [41]	Positive parenting styles	Fix	Yes	*r*=−0.14 (−0.17 to −0.11)	<.001
Niu et al [41]	Negative parenting styles	Fix	Yes	*r*=0.18 (0.14-0.22)	<.001
Zhuang et al [8]	Parent-child relationship	Random	No	*r*=−0.15 (−0.18 to −0.11)	<.05
Social factors					
Noroozi et al [30]	Quality of life	Random	No	OR 0.39 (0.27-0.55)	<.001
Wang et al [38]	Negative life events	Random	Yes	*r*=0.31 (0.28-0.34)	<.001
Wan et al [37]	Social support	Random	Yes	*r*=−0.17 (−0.21 to −0.13)	<.001
Zewde et al [39]	Urban residence	Random	No	OR 2.32 (1.19-4.53)	<.001
Hao et al [42]	Childhood adverse experiences	Random	Yes	*r*=0.21 (0.18-0.24)	<.001
Wu et al [43]	Social anxiety	Random	Yes	*r*=0.34 (0.19-0.48)	<.001

a ES: effect size.

b NS: not significant.

c OR: odds ratio.

On the other hand, the quality assessment conducted using the GRADE tool revealed that Chung’s analyses, which identified a protective impact of the discount probability against digital addiction (*r*=−0.32, 95% CI −0.85 to −0.20; *P*<.47), was graded as high-quality evidence [28]. However, the statistical results were not significant. In addition, Rajesh et al [36] found that different personality traits have varying impacts on digital addiction was also rated as moderate evidence quality. Specifically, they suggested that individuals with openness (*r*=−0.05, 95% CI −0.08 to −0.01; *P*<.05), agreeableness (*r*=−0.07, 95% CI −0.10 to −0.04; *P*<.05), and conscientiousness (*r*=−0.15, 95% CI −0.18 to −0.11; *P*<.001) were protected against the addictive potential of digital media. Conversely, those experiencing loneliness (*r*=0.23, 95% CI 0.19-0.27; *P*<.001), depression (*r*=0.18, 95% CI 0.13-0.23; *P*<.05), impulsivity (*r*=0.25, 95% CI 0.18-0.32; *P*<.001), and shyness (*r*=0.20, 95% CI 0.14-0.25; *P*<.001) are at a greater risk of developing digital addiction. Although no publication bias was found in the above analyses, it is important to emphasize that none of the analyses addressing intrapersonal factors achieved moderate or high ratings in terms of methodology and quality of evidence.

#### Interpersonal Factors

Of the 5 analyses examining interpersonal factors associated with digital addiction, only 1 study by Zhuang et al [8] met the criteria for moderate methodological and evidence quality with AMSTAR 2 tool. This study focused on parent-child relationship and showed that the stronger the parent-child relationship (*r*=−0.15, 95% CI −0.18 to −0.11, *P*<.05), the lower the probability of digital addiction. No risk of publication bias was identified in this analysis (Table 4 and Figure 2).

#### Social Factors

The findings revealed that urban residence (OR 2.32, 95% CI 1.19‐4.53; *P*<.001) by Zewde et al [39], exposure to adverse childhood experiences (*r*=0.21, 95% CI 0.18‐0.24; *P*<.001) by Hao et al [42], and the presence of social anxiety (*r*=0.34, 95% CI 0.19‐0.48; *P*<.001) by Wu et al [43] were significant predictors of digital addiction. Notably, all 3 analyses were accorded moderate ratings in the evaluation of methodological and the quality of evidence rating with AMSTAR 2 and GRADE tools, and no evidence of publication bias was identified in the analyses. Furthermore, Wan et al [37] suggesting that individuals with stronger social support networks (*r*=−0.17, 95% CI −0.21 to −0.13, *P*<.001) exhibited a reduced likelihood of developing digital addiction. However, this particular analysis did not achieve a similar assessment in the quality of evidence, although it was recognized for its moderate methodological quality (Table 4 and Figure 2).

## Discussion

### Principal Findings

This umbrella review identified a range of intra-personal, interpersonal, and social factors influencing digital addiction. In a comprehensive examination of the aforementioned factors, several studies were noted for their methodological assessment. The studies conducted by Ding et al [32] on self-esteem and self-control, by Zewde et al [39] on male gender, and by Wan et al [37] on social support, all achieved moderate rankings for methodological rigor. However, the evidence for these factors was considered circumstantial or questionable in accuracy, resulting in a classification of low quality. On the other hand, study by Zhuang et al [8] concluded that a strong parent-child relationship significantly mitigated digital addiction, while study by Zewde et al [39] identified urban living as a risk factor. In addition, study by Hao et al [42] on childhood adverse experiences, and study by Wu et al [43] on social anxiety, findings from both of these studies received ratings of moderate methodological quality.

Intrapersonal factors significantly influence digital addiction, with our study evaluating 21 such factors, of which only 3 received moderate ratings on the AMSTAR 2 and 8 on the GRADE scale, all focusing on the associations between personality traits and digital addiction. Despite some methodological limitations, our findings were consistent with finding by Geng et al [44], indicating that self-control inversely associated with digital addiction, suggesting that individuals with lower self-regulation are more prone to emotional and impulsive behaviors, thereby fostering harmful digital usage patterns. In addition, specific personality traits can serve as protective factors against digital addiction [44,45]. As previous research has indicated, self-esteem, which is a reflection of an individual’s sense of perceived value, is associated with higher self-efficacy and self-control, ultimately reducing addiction risk [46]. Traits such as self-consciousness, characterized by self-discipline and organizational skills, tend to promote caution in digital interactions, favoring real-life experiences [47]. Furthermore, individuals exhibiting higher levels of agreeableness and openness are less likely to encounter risks associated with digital addiction [36,47]. Conversely, traits such as narcissism, characterized by self-centeredness and lack of empathy, may drive individuals to seek internet-based validation, increasing addiction susceptibility [48]. Consistent with previous research [49-53], impulsivity is a notable risk factor for digital addiction, as highly impulsive individuals often struggle to resist internet-based temptations. Shyness may prompt individuals to engage more in digital socialization to avoid in-person interactions, potentially heightening addiction risk [54]. Thus, our results suggest that while positive personality traits may buffer against digital addiction, negative traits can increase vulnerability. At present, cognitive behavioral therapy and psychosocial interventions are recognized as efficacious strategies for mitigating the symptoms of internet addiction. Cognitive behavioral therapy has been shown to substitute impulsive behaviors with more reasoned actions and improve self-control [55], thereby contributing to the prevention and management of digital addition [56]. Psychosocial interventions have been effective in addressing internet addiction and enhancing self-esteem. By focusing on the psychological and social dimensions of the individual, these approaches promote the development of a positive personality and can be effective in curbing digital addition behaviors [57,58]. Furthermore, gender differences are crucial, as males are more likely to develop digital addictions, likely due to their preference for strategy, role-playing, and action genres, which are inherently more addictive [59].

Among the 4 interpersonal factors associated with digital addiction, 1 study was rated as moderate on both the AMSTAR 2 and GRADE scales, indicating that it provided reasonably convincing evidence through robust methodology [8]. This finding is consistent with previous research, which has demonstrated that enhanced parent-child relationships can effectively reduce the risk of digital addiction among adolescents [60,61]. Research suggests that there is a link between excessive parental screen time and problematic screen behaviors in their offspring. Excessive use of digital devices by parents reduces interaction with their children; this, along with their children’s emulation of their parents’ screen-using habits, contributes to the development of screen-using behaviors in adolescents. This pattern of behavior has the potential to evolve into digital addiction [62,63]. Therefore, providing guidance on the use of digital devices, especially for parents of young children, may become a viable intervention strategy to counteract the emergence of digital addiction in children.

Six of the included studies explored examined the influence of social factors on digital addiction, with 4 rated as moderate quality based on AMSTAR 2 criteria. However, only 3 of these studies received a moderate rating from GRADE. Evidence indicates that urbanization uniquely affects addiction; urban residents often face environmental pollution and discomfort associated with city life, which can heighten psychological stress and contribute to digital addiction [64]. In addition, a lack of social support emerges as a critical risk factor for internet addiction. Insufficient social connections can lead to unhappiness, prompting individuals to seek emotional fulfillment through social media, potentially worsening addiction issues [65,66]. Late childhood, a pivotal period for social and emotional development, shows a marked negative association between internet engagement and various well-being dimensions. This correlation suggests that the rising levels of unhappiness and anxiety in children are closely linked to increased internet use. Furthermore, adverse childhood experiences related to home environments and parental pressures significantly influence children’s development, leading to increased time spent on digital media [[67,68]]. In addition to the previously mentioned parental digital guidance, group counseling has also been reported to play a significant role in enhancing the self-control abilities of school-aged children with internet addiction [69]. However, when parents are also involved in group counseling, research has proven that it requires parents to have previously successfully dealt with challenges such as social anxiety, social isolation, and social skill deficits, or else group counseling will be much less effective [69].

Intrapersonal factors influencing digital addiction have been extensively studied, predominantly focusing on personality traits. However, most meta-analyses seem to suffer from methodological flaws, and even those without serious issues often reveal publication bias in original studies, thereby diminishing the quality of the evidence [32,39]. Therefore, it raises concerns about the risk of publication bias, where positive results are more readily published, potentially obscuring the true nature of the cause-and-effect relationship [70]. In contrast, fewer studies examining interpersonal and social factors demonstrate better methodological rigor and evidence quality. These factors often exert their influence on digital addiction indirectly by affecting psychological traits or personality characteristics. For example, Malak et al [71] used structural modeling to show that social support mediates the relationship between anxiety and stress, contributing to internet-based gaming disorders, a form of digital addiction [66]. Thus, it is misleading to solely consider interpersonal and social factors when exploring correlates of digital addiction. Instead, this indicates the need for further research to elucidate the complex interplay between these domains.

### Limitations

While this umbrella review evaluated numerous factors associated with digital addiction, several limitations persist. First, the focus on published meta-analyses may lead to the omission of factors that are less studied or have not garnered significant attention [72]. Second, the broad scope of the review complicates the generalization of results due to the diversity of research findings included. Finally, many systematic reviews analyzed similar exposure factors and were conducted within a relatively short timeframe, resulting in potential overlap among initial studies. To mitigate this, we prioritized the inclusion of the most recent meta-analyses with the highest number of studies and aimed to exclude those exhibiting considerable overlap with original studies.

### Conclusion

In conclusion, a range of intrapersonal, interpersonal, and social factors significantly influences an individual’s susceptibility to digital addiction. A rigorous evaluation using the AMSTAR and GRADE frameworks identified negative parent-child relationships, urban residence, adverse childhood experiences, and social anxiety as credible risk factors. These findings are essential for informing effective public health strategies aimed at preventing digital addiction. However, further refinement of the methodologies used to analyze these factors is necessary to enhance the robustness of future research.

## Supplementary material

10.2196/66950 Multimedia Appendix 1 Systematic search details in PubMed, Cochrane Library, Scopus, and Web of Science.

## Abbreviations

AMSTAR 2: A Measurement Tool to Assess Systematic Reviews 2
GRADE: Grading of Recommendations Assessment, Development, and Evaluation
JBI checklist: Joanna Briggs Institution Critical Appraisal Checklist
NOS: New Castle-Ottawa scale
OR: odds ratio
PRISMA: Preferred Reporting Items for Systematic Reviews and Meta-Analyses
SSE: small study effect
