# Supplementary material for: Factors Associated With Digital Addiction: Umbrella Review
Source: JMIR Ment Health. 2025 Jul 28;12:e66950. doi: 10.2196/66950 (PMC12303545; doi:10.2196/66950)
Supplement: Multimedia Appendix 1 [file mental-v12-e66950-s001.docx]

**Table S1.** Search strategy for PubMed.

| Step | Strategy |
| --- | --- |
| #1 | digital addiction |
| #2 | digital addiction [Title/Abstract] |
| #3 | digital overuse [Title/Abstract] |
| #4 | internet addiction [Title/Abstract] |
| #5 | online addiction [Title/Abstract] |
| #6 | problematic Internet use [Title/Abstract] |
| #7 | gaming disorder [Title/Abstract] |
| #8 | social media addiction [Title/Abstract] |
| #9 | #1 OR #2 OR #3 OR #4 OR #5 OR #6 OR #7 OR #8 |
| #10 | meta-analysis [Title/Abstract] |
| #11 | systematic review |
| #12 | #10 OR #11 |
| #13 | #9 AND #12 |

**Table S2.** Search strategy for .Web of Science.

| Step | Strategy |
| --- | --- |
| #1 | TS=(digital addiction) OR TI=(digital addiction) OR AB=(digital addiction) |
| #2 | TS=(digital overuse) OR TI=(digital overuse) OR AB=(digital overuse) |
| #3 | TS=(internet addiction) OR TI=(internet addiction) OR AB=(internet addiction) |
| #4 | TS=(online addiction) OR TI=(online addiction) OR AB=(online addiction) |
| #5 | TS=(problematic Internet use) OR TI=(problematic Internet use) OR AB=(problematic Internet use) |
| #6 | TS=(gaming disorder) OR TI=(gaming disorder) OR AB=(gaming disorder) |
| #7 | TS=(social media addiction) OR TI=(social media addiction) OR AB=(social media addiction) |
| #8 | #1 OR #2 OR #3 OR #4 OR #5 OR #6 OR #7 |
| #9 | TS=(meta-analysis) OR TI=(meta-analysis) OR AB=(meta-analysis) |
| #10 | TS=(systematic review) OR TI=(systematic review) OR AB=(systematic review) |
| #11 | #9 OR #10 |
| #12 | #8 AND #11 |

**Table S3.** Search strategy for Cochrane Library.

| Step | Strategy |
| --- | --- |
| #1 | (digital addiction):ti,ab,kw |
| #2 | (digital overuse):ti,ab,kw |
| #3 | (internet addiction):ti,ab,kw |
| #4 | (online addiction):ti,ab,kw |
| #5 | (problematic Internet use):ti,ab,kw |
| #6 | (gaming disorder):ti,ab,kw |
| #7 | (social media addiction):ti,ab,kw |
| #8 | #1 OR #2 OR #3 OR #4 OR #5 OR #6#7 |
| #9 | (meta-analysis):ti,ab,kw |
| #10 | (systematic review):ti,ab,kw |
| #11 | #9 OR #10 |
| #12 | #8 AND #11 |

**Table S4.** Search strategy for Embase.

| Step | Strategy |
| --- | --- |
| #1 | Title, abstract, keywords: digital addiction |
| #2 | Title, abstract, keywords: digital overuse |
| #3 | Title, abstract, keywords: internet addiction |
| #4 | Title, abstract, keywords: online addiction |
| #5 | Title, abstract, keywords: problematic Internet use |
| #6 | Title, abstract, keywords: gaming disorder |
| #7 | Title, abstract, keywords: social media addiction |
| #8 | #1 OR #2 OR #3 OR #4 OR #5 OR #6 OR #7 |
| #9 | Title, abstract, keywords: meta-analysis |
| #10 | Title, abstract, keywords: systematic review |
| #11 | #8 OR #10 |
| #12 | #8 AND #11 |
